# A metal-free and transparent light-emitting device by sequential spray-coating fabrication of all layers including PEDOT:PSS for both electrodes†

**DOI:** 10.1039/d3ra02520a

**Published:** 2023-06-06

**Authors:** Etienne Auroux, Gunel Huseynova, Joan Ràfols-Ribé, Vladimir Miranda La Hera, Ludvig Edman

**Affiliations:** a The Organic Photonics and Electronics Group, Department of Physics, Umeå University SE-90187 Umeå Sweden ludvig.edman@umu.se

## Abstract

The concept of a metal-free and all-organic electroluminescent device is appealing from both sustainability and cost perspectives. Herein, we report the design and fabrication of such a light-emitting electrochemical cell (LEC), comprising a blend of an emissive semiconducting polymer and an ionic liquid as the active material sandwiched between two poly(3,4-ethylenedioxythiophene):poly(styrene-sulfonate) (PEDOT:PSS) conducting-polymer electrodes. In the off-state, this all-organic LEC is highly transparent, and in the on-state, it delivers uniform and fast to turn-on bright surface emission. It is notable that all three device layers were fabricated by material- and cost-efficient spray-coating under ambient air. For the electrodes, we systematically investigated and developed a large number of PEDOT:PSS formulations. We call particular attention to one such p-type doped PEDOT:PSS formulation that was demonstrated to function as the negative cathode, as well as future attempts towards all-organic LECs to carefully consider the effects of electrochemical doping of the electrode in order to achieve optimum device performance.

## Abstract 2

We report on the spray-coating fabrication of a complete metal-free light-emitting electrochemical cell featuring PEDOT:PSS as both electrodes, which is semi-transparent in the off-state and delivers bright emission in the on-state.

## Graphical abstract

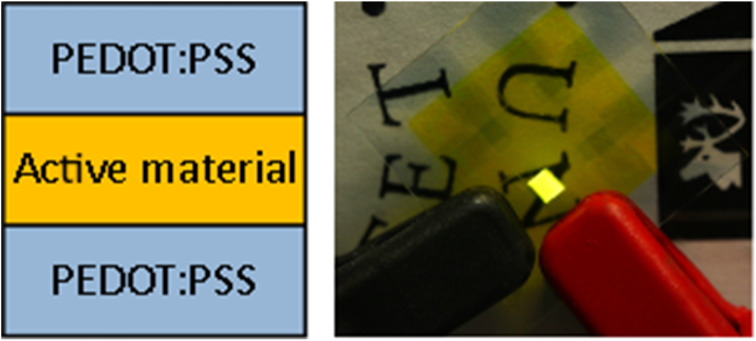

## Introduction

Metals have been a ubiquitous component in electronics because of their high electrical conductivity^1^ since the demonstration of the first diode at the beginning of the last century.^2^ However, the environmental cost of metal extraction and processing,^3,4^ and the difficulties of their recycling,^5^ in combination with that several key metals in electronics, such as In, Pt and the rare earths, are becoming scarce and expensive,^6^ serve as strong motivation for the identification and development of functional alternatives. In this context, the emergence of organic compounds that exhibits high electronic conductivity^7^ is appealing, not the least since they can be synthesized from common organic and even biobased precursors,^8–10^ and since they can be deposited with cost- and energy-efficient methods such as printing and coating.^11^

The organic light-emitting diode (OLED) is a thin-film electronic device that emits light by the process of electroluminescence, and it is – or projected to become – the state of the art in a plethora of applications, spanning from high-end displays, over areal illumination, to transient emissive gadgets.^12–17^ However, from a sustainability perspective, it is a concern that current commercial OLEDs comprise a notably difficult-to-recycle mixture of organic materials and metals, and that they are fabricated by a cost- and material-inefficient vacuum deposition method.^18–21^

The light-emitting electrochemical cell (LEC) is an alternative electroluminescent technology, which is similar in appearance to the OLED but formally distinguished by that the active material that comprises mobile ions. The functional action of these mobile ions during the initial LEC operation has paved the way for fabrication of complete LEC devices with material-efficient coating and printing methods.^22–26^ Moreover, metal-free LECs, comprising chemically derived graphenes^27,28^ or carbon nanotubes^29–31^ for the electrodes, have been reported, but the drawbacks were that the graphene conversion required exposure to a reducing atmosphere at a high temperature of 1000 °C, whereas the carbon nanotube deposition included a difficult-to-scale filtration, stamping and lamination process.

Herein, we report on the benign and scalable fabrication of a completely metal-free LEC, featuring a conducting polymer, p-type doped poly(3,4-ethylenedioxythiophene):poly(styrene-sulfonate) (PEDOT:PSS), for both electrodes. It is notable that all three device layers were deposited by scalable and resource-efficient spray-coating under ambient air, that the all-organic LEC features a high transparency in the off-state, and that it delivers a bright and uniform luminance of 610 cd m^−2^ in the on-state. Our study thus demonstrates that metal-free and all-solution-processed emissive devices can become a viable option in the future, but also brings forward interesting and important questions as regards the fundamental operation and rational design of these devices for practical applications.

## Experimental

### The PEDOT:PSS inks and films

Five different pristine “PEDOT:PSS-*x*”, with *x* ranging between 1 and 5, inks were obtained from Heraeus (GER), and their herein employed abbreviations (with their trade names in parentheses) are as follows: PEDOT:PSS-1 (Clevios P.VP.AI 4083), PEDOT:PSS-2 (Clevios PH1000), PEDOT:PSS-3 (Clevios P.HY.E), PEDOT:PSS-4 (Clevios SV3) and PEDOT:PSS-5 (Clevios SV4). The “PEDOT:PSS-*x-m*” inks were prepared by addition of 400 vol% methanol (VWR, GER) to the corresponding pristine ink under ambient air, followed by sonication for >45 min in an ultrasonic bath (VWR, GER). The “PEDOT:PSS-*x-w*” inks were prepared by addition of 400 vol% distilled water to the corresponding pristine ink under ambient air, followed by sonication for >45 min in the ultrasonic bath.

The glass substrates (Kintec, CHN) were cleaned by sequential 15 min ultrasonic treatment in distilled water, acetone (VWR, GER), and isopropanol (VWR, GER), and thereafter dried at 120 °C for ≥12 h. The more concentrated and viscous pristine PEDOT:PSS-*x* inks were deposited on the cleaned glass substrates by either spin-coating at 2000 rpm for 60 s (*x* = 1, 2, and 3) or by blade-coating (*x* = 4 and 5), and thereafter dried at 100 °C for 20 min on a hotplate. The diluted and therefore less viscous PEDOT:PSS-*x-m* and PEDOT:PSS-*x-w* inks were spray-coated onto the cleaned glass substrates, positioned on a hot plate at 80 °C, using a computer-controlled spray box (LunaLEC, SWE) equipped with an internal-mix spray nozzle. The spray nozzle was moving on a planar stage 9 cm above the glass substrate, and the spray parameters were: N_2_ gas pressure = 410 kPa, ink flow rate = 4 ml min^−1^, number of sweeps = 3, spray time = 60 s. The spray-coated films were dried on a hotplate at 80 °C for >1 h (the water-containing inks) or at 120 °C for 4 min (the methanol-containing inks). The dry PEDOT:PSS films were either used as is, or immersed into a dimethyl sulfoxide (DMSO) bath for 30 min and then dried at 180 °C for 10 min on the hotplate, following a procedure reported in the literature.^32^ The latter films are termed d-PEDOT:PSS-*x-y* (with *y* being equal to *m* or *w*, as described above).

The thickness of the PEDOT:PSS films was determined using a stylus profilometer (Dektak XT, Bruker, US) and varied from 80 nm for the thinnest spin-coated film to 1200 nm for the thickest blade-coated film. The sheet resistance (*R*_S_) of the PEDOT:PSS films was measured using a four-point probe setup (HM21 Jandel, Bridge Technology, US). The combination of *R*_S_ and the film thickness enabled the calculation of the conductivity of the PEDOT:PSS films. The transmittance of the PEDOT:PSS films was measured using a spectrophotometer (Lambda 1050, PerkinElmer, USA). The combination of the transmittance and the film thickness enabled the determination of the absorption coefficient of the PEDOT:PSS films using the Beer–Lambert law.

The work function of the PEDOT:PSS films was determined by ultraviolet photoelectron spectroscopy (UPS). The UPS setup consisted of a UV light source (He gas photon energy = 21.212 eV) and a hemispherical electron analyzer (Scienta-R3000, VG SCIENTA) positioned in a vacuum chamber (*p* < 1 × 10^−10^ mbar). The UPS spectrum was measured with an acceptance angle of 6°, a pass energy of 10 eV, a slit width of 1.3 mm, and an energy step of 1.99 meV. The measurement resolution of the UPS is 4.8 meV and the values for the binding energy were derived with respect to the Fermi level. The work function was determined from the secondary electron cut-off spectra recorded with a bias of −7 V.

### Device fabrication and measurement

The all-organic devices were fabricated by spray-coating under ambient air using the spray box (LunaLEC, SWE). The PEDOT:PSS-2-*m* ink was spray-coated onto a cleaned glass substrate through a shadow mask, which defined four bottom electrodes on one substrate. The spray nozzle was moving on a planar stage 9 cm above the glass substrate, and the spray parameters are as follows: N_2_ gas pressure = 410 kPa, ink flow rate = 4 ml min^−1^, number of sweeps = 4, spray time = 80 s. Directly after the spray-coating of the PEDOT:PSS-2-*m* bottom electrodes, the substrate was placed on a hotplate at 120 °C for ≥4 min. The dry thickness of the PEDOT:PSS-2-*m* bottom electrode was 170 nm, as measured using a stylus profilometer.

Poly(methyl methacrylate) (PMMA, powder, Merck, GER) was dissolved in butyl acetate (Merck, GER) in a 20 g l^−1^ concentration. This solution was stirred at 70 °C for >24 h in a N_2_-filled glovebox ([O_2_] < 1 ppm, [H_2_O] < 1 ppm), and thereafter diluted with 450 vol% ethyl acetate (VWR, GER) under ambient air for the formulation of the PMMA ink. The spray box was employed to spray-sinter the PMMA ink through a shadow mask for the formation of an electrically insulating “patterning layer”, which further defined the subsequent device emission area as the PMMA-free PEDOT:PSS. The PMMA spray-sintering was performed with the substrate positioned on a hot plate at 80 °C under ambient air. The nozzle was moving 6 cm above the substrate, and the spray parameters are as follows: N_2_ gas pressure = 450 kPa, ink flow rate = 4 ml min^−1^, number of sweeps = 6, spray time = 120 s. The dry thickness of the insulating PMMA pattering layer was 650 nm, as measured using a stylus profilometer.

The active material comprised a blend of a conjugated poly(paraphenylene vinylene) co-polymer termed Super Yellow (catalogue number: PDY-132, Merck, GER) and the ionic liquid tetrahexylammonium tetrafluoroborate (THABF_4_, Merck, GER) for the all-organic LEC and solely Super Yellow for the all-organic OLED. Super Yellow and THABF_4_ were separately dissolved in cyclohexanone in 8 g l^−1^ and 10 g l^−1^ concentration, respectively, in a N_2_-filled glovebox ([O_2_] < 1 ppm, [H_2_O] < 1 ppm). These two master solutions were stirred for >24 h at 70 °C, and thereafter blended in a THABF_4_:Super Yellow mass ratio of 0.08 : 1. The blend solution was stirred at 70 °C for >2 h and thereafter diluted with 450 vol% tetrahydrofuran (THF, Merck, GER) under ambient air for the formulation of the active-material ink. The active-material ink was spray-sintered^33^ on top of the bottom-PEDOT:PSS-electrode/PMMA-patterning-layer under ambient air, with the substrate kept at 80 °C by the hot plate in the spray box. The nozzle was moving 6 cm above the substrate and the spray parameters are as follows: N_2_ gas pressure = 450 kPa, ink flow rate = 4 ml min^−1^, number of sweeps = 6, spray time = 120 s. The dry active-material thickness was 300 nm, as measured using a stylus profilometer.

A second insulating PMMA patterning layer was spray-sintered on top of the active material using the PMMA ink and spray parameters of the first PMMA layer. The PMMA ink was deposited through a shadow mask to spatially define the PEDOT:PSS top electrode (by the absence of PMMA). The thickness of the upper insulating PMMA patterning layer was 650 nm, as measured using a stylus profilometer.

The PEDOT:PSS-4-*m* ink was spray-coated onto the top of the active material/PMMA assembly through a shadow mask, which defined four top PEDOT:PSS-4-*m* electrodes on each substrate. The spray nozzle was moving on a planar stage 9 cm above the glass substrate, and the spray parameters are as follows: N_2_ gas pressure = 410 kPa, ink flow rate = 4 ml min^−1^, number of sweeps = 3, spray time = 60 s. Directly after the spray-coating of the PEDOT:PSS-4-*m* top electrodes, the substrate was placed on a hotplate at 120 °C for ≥4 min. The dry thickness of the PEDOT:PSS-4-*m* top electrode was 350 nm, as measured using a stylus profilometer. The overlap and direct contact between the top and bottom electrodes and the active material defined four identical 2 × 2 mm^2^ all-organic devices on each substrate. A schematic of the device structure can be found in Fig. S1.†

The all-organic LECs and OLEDs were electrically driven and measured using a source measure unit (Keithley 2400 Source Meter, Tektronix, US), with the driving voltage set to 40 V and the current compliance to 50 mA. The luminance was measured using a photodiode, equipped with an eye-response filter (BPW 21, Osram Semiconductors), which had been calibrated using a luminance meter (Konica Minolta LS-110). The device characterization was performed in the glovebox.

## Results and discussion

The principal goal of the study was to fabricate a complete electroluminescent device by spray-coating under ambient air, using solely metal-free and organic compounds for all device layers. Such complete solution-based deposition is challenging, in part because the solution-based deposition of a wet upper layer can dissolve and/or damage the solution-processed layer below. We herein address this challenge by designing and employing a robust and relatively simple three-layer device structure, comprising an all-hydrophobic active material sandwiched between two hydrophilic electrodes. The active material comprised a hydrophobic electroluminescent conjugated polymer termed Super Yellow blended with a hydrophobic THABF_4_ ionic liquid as the electrolyte, while the two electrodes were fabricated from two carefully tuned hydrophilic poly(3,4-ethylenedioxythiophene):poly(styrene sulfonate) (PEDOT:PSS) inks. In addition, we selected to include non-essential poly(methyl methacrylate) (PMMA) “patterning layers” in between the electrodes and the active material for better performance reproducibility (see Fig. S1†).

PEDOT:PSS is a very interesting and unique material in that it can be formulated into inks, which can be cast into thin films that feature an attractive combination of high conductivity and high transparency. The PEDOT:PSS inks can be tuned by a variety of methods and additives, in order to make their properties fit and promising for different applications.^34–37^ The selection and modification of the PEDOT:PSS inks and the corresponding solute materials for the device electrodes were performed in consideration of the following five goal criteria: (i) the ink shall enable solution-based fabrication of uniform thin films by spray-coating; (ii) the deposited film shall leave the beneath layers intact and exhibit an intimate electronic contact with the neighboring active-material film; (iii) the dry PEDOT:PSS film shall exhibit a conductivity of at least 100 S cm^−1^, (iv) the transparency of the dry PEDOT:PSS film in the visible range shall be at least 70%, and (v) it must be metal free.

The conductivity requirement was derived with the ambition of enabling uniform light emission from the entire device area. This implied that the “lateral” sheet resistance of the PEDOT:PSS electrode shall be negligible, *i.e.*, <10%, compared to the effective “vertical” steady-state resistance of the active material. The value for this effective steady-state resistance of the active material of 10 kΩ was gleaned from previous studies on identically sized devices comprising the same Super Yellow:THABF_4_ active material positioned between Al and indium-tin-oxide (ITO) as the electrodes.^38,39^ Therefore, a 100 nm thick PEDOT:PSS film should exhibit a conductivity of at least 100 S cm^−1^. A high visible-range transparency is an obvious requirement for the efficient outcoupling of the light generated within the active material to an outside observer, as well as an enabler for the realization of a transparent light source.

In this study, we identified the following five “pristine PEDOT:PSS-*x*” inks (with their trade names in parentheses) for investigation and modification: PEDOT:PSS-1 (Clevios P.VP.AI 4083), PEDOT:PSS-2 (Clevios PH1000), PEDOT:PSS-3 (Clevios P.HY.E), PEDOT:PSS-4 (Clevios SV3) and PEDOT:PSS-5 (Clevios SV4). The pristine PEDOT:PSS-*x* inks were deposited as thin films by either spin-coating (PEDOT:PSS-1,2,3) or blade-coating (PEDOT:PSS-4,5), because they were found to be too viscous for deposition by our preferred method of spray-coating. We therefore diluted the pristine PEDOT:PSS-*x* inks with 400 vol% of either methanol or water for the formulation of PEDOT:PSS-*x-m* and PEDOT:PSS-*x-w* inks, respectively. The motivation for selecting water is that it is already (part of) the solvent of the pristine PEDOT:PSS-*x* inks, while methanol is a common conductivity enhancer of PEDOT:PSS.^40–42^ This selection was further motivated by water and methanol being desirable solvents from both safety and environmental perspectives.^43^ Some of the dry films were finally immersed into a potentially conductivity-enhancing^32^ dimethyl sulfoxide (DMSO) bath for 30 min and then dried at 180 °C for 10 min; these films are identified by the addition of the letter d before their name.

**Fig. 1:**
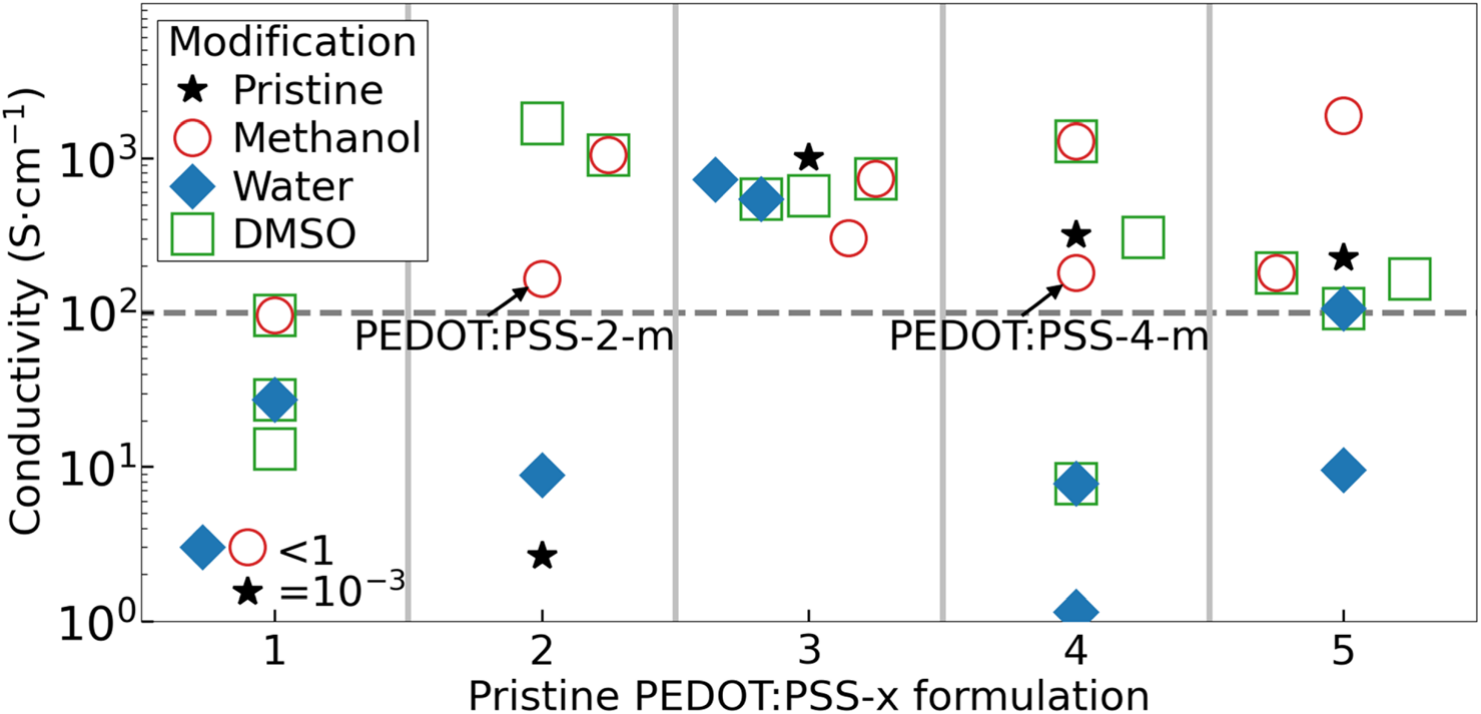
Conductivity of the solution-processed PEDOT:PSS-*x-y* thin films on glass substrates, with the *x* value of the pristine ink formulation identified by the *x*-axis value and the ink modification *y* identified in the inset. The pristine PEDOT:PSS-*x* inks were either spin-coated (*x* = 1, 2, and 3) or blade-coated (*x* = 4 and 5). The PEDOT:PSS-*x-m* and PEDOT:PSS-*x-w* inks were modified by dilution with methanol and water, respectively, and deposited by spray-coating. The d-PEDOT:PSS-*x-y* films were prepared by immersion of the dry PEDOT:PSS-*x-y* film into a DMSO bath for 30 min.

Fig. 1 presents the measured conductivity of the thin films fabricated from the pristine and modified PEDOT:PSS ink formulations. For PEDOT:PSS-1, we found that all of the inks resulted in a dry film conductivity significantly or slightly below our derived threshold of 100 S cm^−1^, as indicated by the horizontal dashed grey line in Fig. 1. The PEDOT:PSS-1 inks were therefore dismissed from further investigation. The pristine and water-modified PEDOT:PSS-2 films also featured an insufficient conductivity, but the addition of methanol and in particular the (subsequent) treatment of DMSO resulted in the attainment of a high conductivity of up to ∼1000 S cm^−1^. All of the dry PEDOT:PSS-3 films exhibited a very high conductivity, which was highly independent on the modification procedure. This is rationalized by that the pristine ink comprises highly conducting Ag nanowires. However, since this inclusion is a drawback from a metal and sustainability point of view, the PEDOT:PSS-3 inks were disqualified from further evaluation. For the PEDOT:PSS-4 and PEDOT:PSS-5 inks, the trend is similar in that both the pristine, methanol and DMSO-modified inks delivered a sufficiently high film conductivity, while the addition of water to the ink resulted in a significant lowering of the film conductivity.

We remind that all five pristine PEDOT:PSS-*x* inks were non-fit for the purpose of this study, *i.e.*, deposition by spray-coating, since their high viscosity prohibited an efficient ink passage through the spray nozzle without clogging. Moreover, the water-modified PEDOT:PSS-*x-w* inks were found to invariably require a very long drying time of >1 h at 80 °C, since the high boiling point (low vapor pressure) of water resulted in the formation of a very wet film on the substrate. Our attempts with a higher drying temperature of ≥100 °C were unsuccessful because it caused vibrant boiling of the remnant water in the wet film on the substrate, which resulted in dry PEDOT:PSS-*x-w* films being non-uniform and damaged. In contrast, the spray-coating of the methanol-modified PEDOT:PSS-*x-m* inks resulted in the formation of a much drier film on the substrate, since a large fraction of the low-boiling-point methanol majority solvent had evaporated already during the transfer of the spray droplets from the nozzle to the substrate.^33^ This enabled a fast drying process consuming 4 min at 120 °C for the formation of uniform PEDOT:PSS-*x-m* films. The exception was the PEDOT:PSS-5-*m* ink, which formed a film of poor quality following spray-coating. Finally, the DMSO modification was generally successful from a conductivity perspective, but found to suffer severely from a film quality perspective because of edge damages and poor film uniformity.

**Fig. 2:**
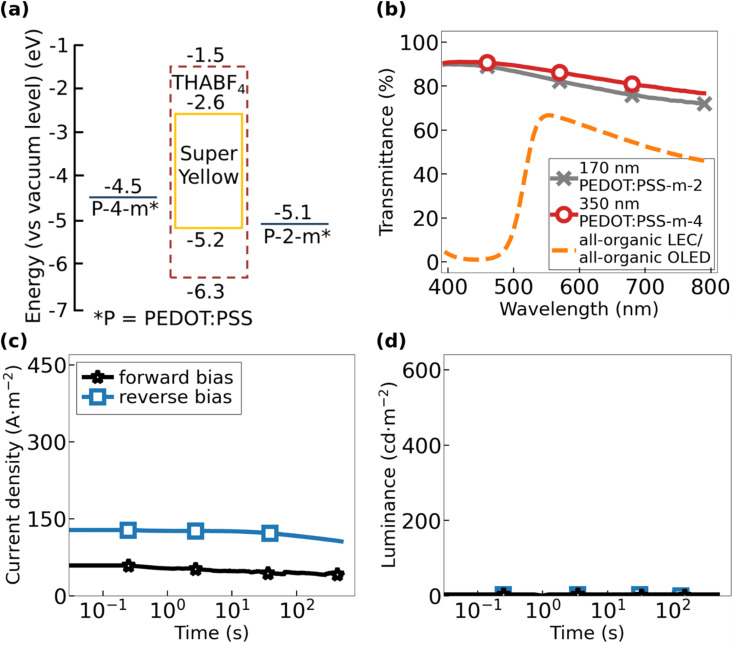
(a) Electron-energy diagram of the all-organic OLED and the all-organic LEC, with the two PEDOT:PSS-2-*m* and PEDOT:PSS-4-*m* electrodes sandwiching the active material comprising the organic semiconductor Super Yellow. The LEC is distinguished from the OLED by that a THABF_4_ electrolyte is blended with Super Yellow in the active material, and its electrochemical stability window is indicated by the dashed brown line. (b) Visible-range transmittance of the 170 nm thick PEDOT:PSS-2-*m* electrode (grey crosses), the 350 nm PEDOT:PSS-4-*m* electrode (open red circles), and the complete all-organic OLED and the complete all-organic LEC in their pristine off-state (dashed orange line). The temporal evolution of (c) the current density and (d) the forward luminance of the all-organic OLED during electric driving by 40 V in “forward bias” with PEDOT:PSS-2-*m* being the negative cathode (black stars) and in reverse bias with the PEDOT:PSS-4-*m* electrode being the negative cathode (open blue squares).

Thus, in summary, our performance and sustainability evaluation revealed that only two of the investigated inks, namely, PEDOT:PSS-2-*m* and PEDOT:PSS-4-*m*, fulfill the five qualification criteria, as defined above. More specifically, these two metal-free inks can be spray-coated as uniform thin films (with short drying time), while leaving the below layers in LEC devices intact. The conductivity of a spray-coated thin film of PEDOT:PSS-2-*m* is 165 S cm^−1^, while that of PEDOT:PSS-4-*m* is 180 S cm^−1^. Fig. 2(b) further shows that spray-coated thin films of PEDOT:PSS-2-*m* (thickness = 170 nm) and PEDOT:PSS-4-*m* (thickness = 350 nm) exhibit a transparency well above 70% over the entire visible wavelength region.

Fig. 2(a) presents the electron–energy diagram of the all-organic OLED and all-organic LEC, both comprising Super Yellow as the emissive organic semiconductor and PEDOT:PSS-2-*m* and PEDOT:PSS-4-*m* as the two electrodes. The two devices are solely distinguished by that the LEC comprises the ionic liquid THABF_4_ as the source of mobile ions in the active material. The values for the highest occupied molecular orbital (HOMO) and the lowest unoccupied molecular orbital (LUMO) of Super Yellow (solid yellow line) as well as the electrochemical stability window of THABF_4_ (dashed brown line) were taken from the literature.^44–47^ Importantly, the THABF_4_ electrolyte is electrochemically inert over the voltage range spanned by the HOMO and LUMO levels of Super Yellow, and therefore, it should not cause unwanted side reactions during the electrochemical operation of the all-organic LEC.^48^

We performed ultraviolet photoelectron spectroscopy (UPS) to measure the work function of the two PEDOT:PSS electrodes. We found that the two compounds are deviating significantly in this regard, as the work function of PEDOT:PSS-2-*m* is 5.1 eV and that of PEDOT:PSS-4-*m* is 4.5 eV, but note that both values are within the reported range for the work function of different formulations of PEDOT:PSS.^49–53^ Since the lowering of the work function of a p-doped semiconductor is concomitant with a lowering of the p-doping concentration, this result implies that PEDOT:PSS-4-*m* exhibits a significantly lower p-doping concentration than that of PEDOT:PSS-2-*m*. Considering further that conductivity is the product of the doping concentration and the (average) mobility, and the observation that the PEDOT:PSS-2-*m* and PEDOT:PSS-4-*m* films exhibit a highly similar conductivity (see Fig. 1), we draw the conclusion that the hole mobility is much higher for PEDOT:PSS-4-*m* than for PEDOT:PSS-2-*m*.

Fig. 2(b) presents the, essentially identical, transmittance as a function of wavelength for the complete all-organic OLED stack and the complete all-organic LEC stack in the non-biased off-state (dashed orange line). We found that both devices exhibit a transparency exceeding 50% in between 525 and 750 nm, and mention that the absorption at shorter wavelengths in the blue regime is primarily due to the organic semiconductor Super Yellow.^54^ The highly transparent nature of both PEDOT:PSS electrodes (grey crosses and open red circles) is attractive in that it allows for the realization of a see-through device, in particular if a more transparent compound is selected for the organic semiconductor; this device will in addition emit with the same intensity from both planar sides.

Fig. 2(c) and (d) present the transients for the current density and the forward luminance, respectively, for the all-organic OLED during driving with a constant voltage of 40 V. The “forward bias” direction was arbitrarily selected as with PEDOT:PSS-4-*m* being the positive anode and PEDOT:PSS-2-*m* being the negative cathode (black stars), and *vice versa* for “reverse bias”. Two consistent observations for all investigated all-organic OLEDs are that the current density is relatively constant during the entire measurement period and that no light emission is observed, regardless of whether the device is operated in forward or reverse bias (see also Fig. S2 and S3†).

The observed lack of light emission from the all-organic OLED was attributed to the large energy difference of 1.9–2.5 eV between the Fermi level of the negative PEDOT:PSS cathode and the LUMO level of Super Yellow (see Fig. 2(a)), which effectively prohibits electron injection. Such a lack of electron injection will obviously also eliminate the possibility for exciton formation in the active material of the all-organic OLED. The measured current is therefore solely a hole current. This conclusion is supported by the fact that the measured current density is higher in reverse bias than in forward bias, since the energy barrier for hole injection from PEDOT:PSS to the HOMO of Super Yellow is markedly lower at 0.1 eV with PEDOT:PSS-2-*m* as the positive anode compared to 0.7 eV for PEDOT:PSS-4-*m*.

**Fig. 3:**
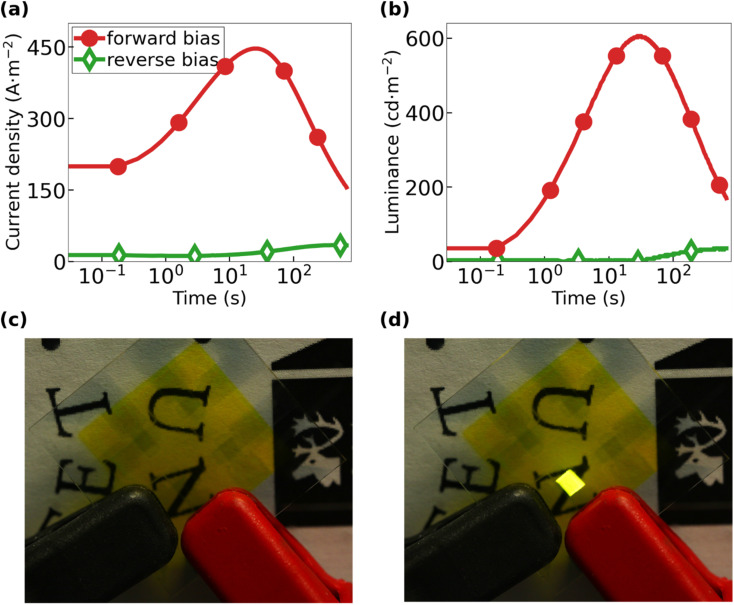
Temporal evolution of (a) the current density and (b) the forward luminance of the all-organic LEC during electric driving with 40 V, in either forward bias with PEDOT:PSS-2-*m* being the negative cathode (solid red circles) or in reverse bias with PEDOT:PSS-4-*m* electrode being the negative cathode (open green diamonds). Photographs of the all-organic LEC (c) in the transparent off-state with no applied voltage and (d) during light emission with 40 V applied in forward bias.

Fig. 3(a) and (b) present the transients for the current density and the forward luminance, respectively, for the all-organic LEC during 40 V constant-voltage driving. The all-organic LEC was operated in either “forward bias” with the PEDOT:PSS-2-*m* electrode biased as the negative cathode (solid red circles) or in “reverse bias” with the PEDOT:PSS-4-*m* electrode biased as the negative cathode (open green diamonds). The forward-biased all-organic LEC exhibits a marked increase of both the current density and the luminance with time, with the peak value for the current density being 445 A m^−2^ and the peak luminance being 610 cd m^−2^. It is notable that the all-organic LEC delivers a much lower current density and essentially no light emission in reverse bias. This forward- and reverse-bias performance of the all-organic LEC is highly repeatable as shown in Fig. S4 and S5.†

A strong increase in the current density during constant-voltage operation is a characteristic feature of functional LEC devices, since it signals an initial injection-facilitating electric double-layer formation at the two electrode interfaces and a subsequent transport-facilitating electrochemical doping of the Super Yellow organic semiconductor. The electrochemical doping takes place at the two electrodes and is p-type at the positive anode and n-type at the negative cathode. These two doping regions eventually make contact under the formation of a p–n junction doping structure.^55,56^ The electric double-layer formation is particularly important at the cathodic interface in our all-organic LECs, since it enables the efficient injection of electrons by tunneling despite the large energy barrier height at this interface (see Fig. 2(a)).

The temporal increase in the luminance is the combined effect of the increasing current density, the improving balance in the electron and hole injection, and the increasing probability of electron and hole recombination during the p–n junction formation.^57–59^ The electron-to-photon transformation can be quantified by the current efficacy, which is 1.4 cd A^−1^ for the forward-biased all-organic LEC at peak luminance. This value is in good agreement with previous reports for this particular active material.^38,39,60^Fig. 3(c) and (d) are photographs of the all-organic LEC in the off-state with no applied bias and in the on-state when the applied forward bias results in uniform and bright light emission, respectively. Importantly, this set of observations demonstrate that it is indeed possible to fabricate a completely metal-free light source by sequential spray-coating under ambient air, which in addition features the interesting property of being semitransparent in the off-state.

With this attractive opportunity established, we finish by drawing attention to three, at first glance, puzzling observations. First, the all-organic LEC features a much higher current density in forward bias than in reverse bias (see Fig. 3(a), S4a and S5a†), despite that the only asymmetry from an energy-level perspective is that the injection barriers are higher in forward bias (see Fig. 2(a)). Second, the all-organic LEC only delivers significant light emission when driven by a forward bias (Fig. 3(b), S4b and S5b†). Third, the drive voltage of 40 V required to generate significant light emission (in forward bias) is much higher than that of LECs based on the same active material but equipped with a “hard” metal instead of “soft” PEDOT:PSS for the negative cathode.^38,39^

In this context, we call attention to that it has been recently demonstrated that a significant fraction of the negative anions in the active material can transfer into a positive PEDOT:PSS anode during the initial LEC operation because of its soft and porous nature, and that this anion transfer can result in additional (electrochemical) p-type doping of the already p-type doped PEDOT.^38^ It seems reasonable that a similar influx of positive cations into a negative soft PEDOT:PSS cathode can take place during the initial LEC operation, but that the corresponding electrochemical reaction should then be reduction or undoping of the p-type doped PEDOT. Such an electrochemical undoping process of the negatively biased PEDOT:PSS cathode has the undesired consequence that its conductivity will decrease during the initial LEC operation, which, in turn, explains why the all-organic LEC required such a high-drive voltage. The fact that the all-organic LEC delivered a higher current and luminance in forward bias than in reverse bias can then be rationalized by that the PEDOT:PSS-2-*m* electrode exhibits a much higher p-type doping concentration than that of the PEDOT:PSS-4-*m* electrode, as concluded from the analysis of the UPS data in Fig. 2(a). PEDOT:PSS-2-*m* should accordingly be able to “absorb” more electrochemical undoping without severely losing its conductivity when biased as the negative cathode than PEDOT:PSS-4-*m*. An important take-home message for the future development of all-organic LECs is then that it is advisable to identify and employ an n-type doped organic semiconductor^61^ for the negative cathode in order to attain optimum device performance.

## Conclusions

We demonstrated that a functional light-emitting device can be fabricated from entirely metal-free organic materials by time- and resource-efficient spray-coating under ambient air. This breakthrough was enabled by the identification and appropriate tuning of two different PEDOT:PSS formulations, which were utilized for the spray-coating of the positive anode and the negative cathode. We further showed that it is the redistribution of the mobile ions in the active material of the LEC that enables the injection of electrons from the PEDOT:PSS cathode into the active material and the associated light emission, but that this ion redistribution can also result in conductivity-decreasing de-doping of the p-type doped PEDOT. The presented all-organic LEC features more than 50% transmittance over a large portion of the visible wavelength range in the off-state and delivers bright and uniform luminance of 610 cd m^−2^ in the on-state. Importantly, our study demonstrates that it is possible to fabricate metal-free light-emitting devices with high-throughput printing and coating methods under ambient air.

## Data availability

All relevant data are available from the corresponding authors upon request.

## Author contributions

L. E., G. H. and E. A. conceptualized the idea. E. A., G. H., J. R. R. and V. M. H. performed the experimental work. All authors contributed to the data analysis. L. E. supervised the project and acquired the funding. L. E. and E. A. wrote the manuscript. All authors have reviewed and approved the final version of the manuscript.

## Conflicts of interest

The authors declare that they have no conflict of interest.

## Supplementary Material

RA-013-D3RA02520A-s001
