# Supplementary material for: A metal-free and transparent light-emitting device by sequential spray-coating fabrication of all layers including PEDOT:PSS for both electrodes
Source: RSC Adv. 2023 Jun 6;13(25):16943–51. doi: 10.1039/d3ra02520a (PMC10242295; doi:10.1039/d3ra02520a)
Supplement: RA-013-D3RA02520A-s001 [file RA-013-D3RA02520A-s001.pdf]

Electronic Supplementary Material (ESI) for RSC Advances.
This journal is © The Royal Society of Chemistry 2023

Supplementary Information:

A metal-free and transparent light-emitting device by sequential spray-coating fabrication of all layers including PEDOT:PSS for both electrodes

Etienne Auroux, Gunel Huseynova, Joan Ràfols-Ribé, Vladimir Miranda La Hera and Ludvig Edman*

The Organic Photonics and Electronics Group, Department of Physics, Umeå University, SE-90187 Umeå, Sweden

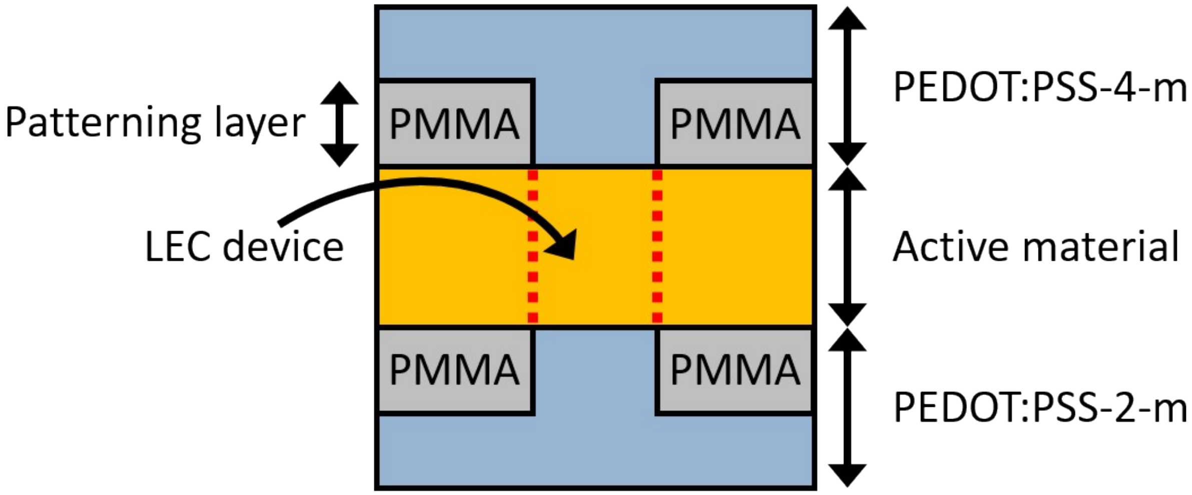

Figure S1. *The device structure of the all-organic LEC.*

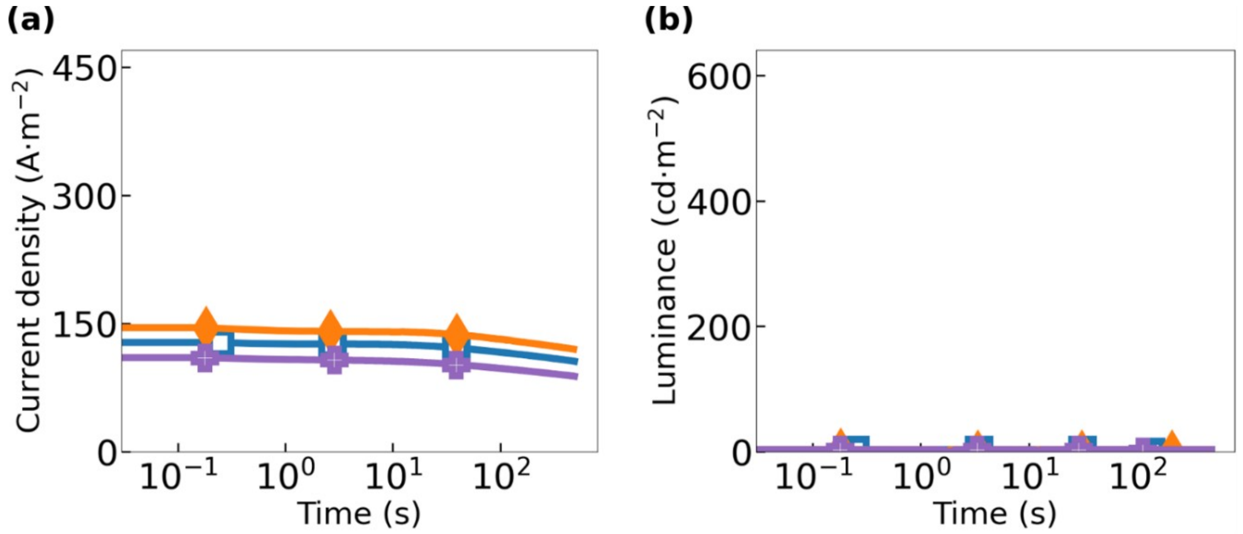

Figure S2. *The temporal evolution of (a) the current density and (b) the luminance of three all-organic OLEDs featuring a 350 nm thick PEDOT:PSS-4-m cathode, a 300 nm thick SY active material, and a 170 nm thick PEDOT:PSS-2-m anode during 40 V constant voltage driving in "reverse bias".*

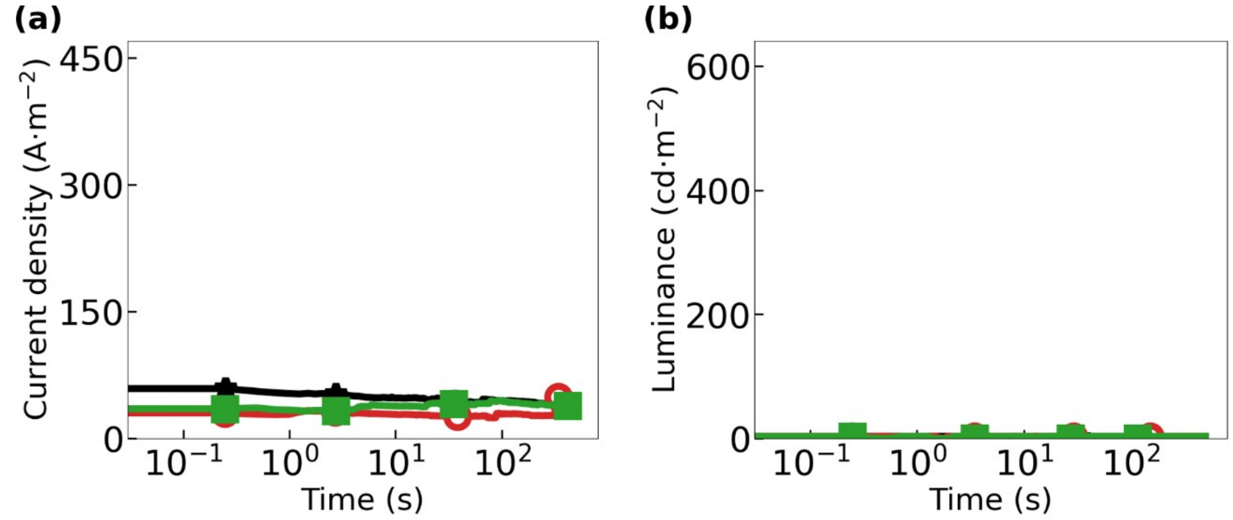

Figure S3. *The temporal evolution of (a) the current density and (b) the luminance of three different all-organic OLEDs featuring a 170 nm thick PEDOT:PSS-2-m cathode, a 300 nm thick Super Yellow active material, and a 350 nm thick PEDOT:PSS-4-m anode during 40 V constant voltage driving in "forward bias".*

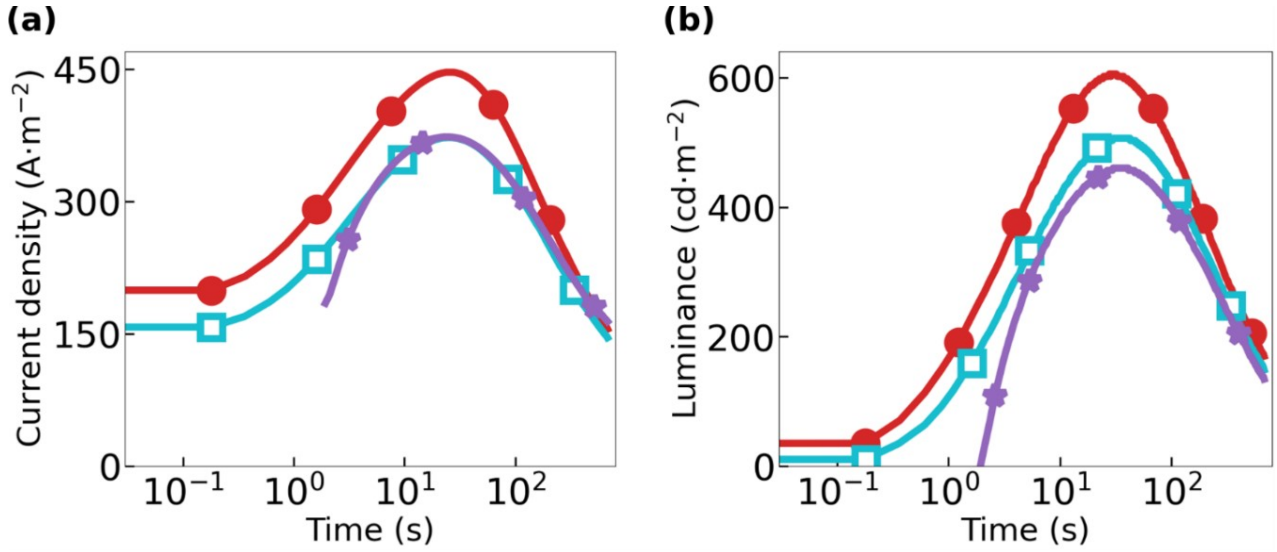

Figure S4. *The temporal evolution of (a) the current density and (b) the luminance for three all-organic LECs driven by a "forward bias" of 40 V. The device features a 170 nm thick PEDOT:PSS-2-m cathode, a 300 nm thick THABF*$_4$*:SY = 8 mass% active material, and a 350 nm thick PEDOT:PSS-4-m anode.*

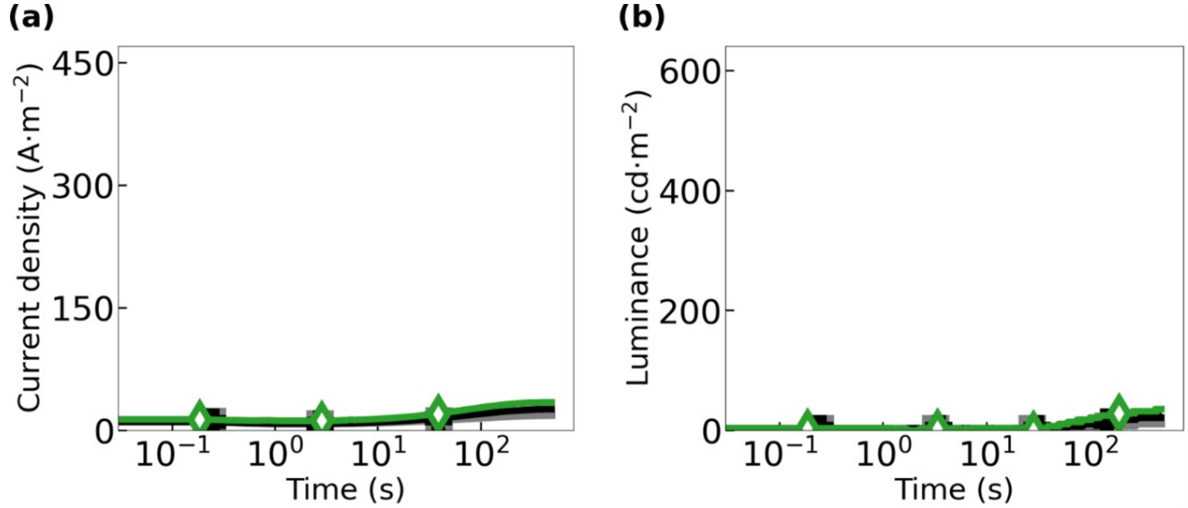

Figure S5. *The temporal evolution of (a) the current density and (b) the luminance of the three all-organic LECs during driving in "reverse bias" of 40 V. The devices feature a 350 nm thick PEDOT:PSS-4-m cathode, a 300 nm thick THABF*$_4$*:SY = 8 mass% active material, and a 170 nm thick PEDOT:PSS-2-m anode.*
